# Promoting family planning use after childbirth and desire to limit childbearing in Ethiopia

**DOI:** 10.1186/1742-4755-11-53

**Published:** 2014-07-16

**Authors:** A Sathiya Susuman, Aristide Bado, Yishak Abraham Lailulo

**Affiliations:** 1 Department of Statistics & Population Studies, University of the Western Cape, Cape Town, South Africa

**Keywords:** Family planning knowledge, Contraceptive use, Desired family size, Spacing, Limiting, Fertility, Mortality

## Abstract

**Background:**

In Ethiopia the average fertility rate in rural areas is about 6 children per woman, while it is 2.4 children per woman in urban areas. It is with this concept in mind that the investigators of this study wanted to correlate the promotion of after-child-birth-use of family planning and desire to limit childbearing in Ethiopia. Postpartum amenorrhea signifies the interval between childbirth and the return of menstruation.

**Objectives:**

The specific objective is to examine the desire to limit family size, along with cases of sterilized, fecund, postpartum amenorrhoea, declared in-fecund and menopausal women within the study area.

**Methods:**

The study is based on the analysis of secondary data obtained from the 2011 Ethiopian Demographic and Health Survey (EDHS). This study is concentrated on couples because we need to know more about married people’s desire to limit their family size. The bivariate, ANOVA, and multivariate analyses were used to analyse the association.

**Results:**

The total number of respondents was 6,745 (78.3% rural and 21.7% urban), with 93.6% of them being currently married and 6.4% of them living with a partner. The mean duration of amenorrhea among women who gave birth in the five years preceding the survey is 16 months. Women with equal numbers of sons and daughters were found to be 75.4% (OR = 0.25) less likely to desire more children, compared to women with more sons than daughters.

**Conclusion:**

Achievable resolutions include increasing females’ ages at marriage, avoiding unwanted teenage pregnancies, completely eradicating home delivery, and inspiring young people to use modern methods of family planning to achieve Millennium Development Goals 4 & 5.

## Background

About 46% of the Ethiopian population is under the age of 15, and 6% is over the age of 60, implying a high dependency ratio [1]. The size and growth of the population, though not a problem by itself, can become a problem when it is not balanced with the available resources and developmental pace of the country [2-6]. Studies in a number of countries have shown that wherever fertility is high, maternal, infant, and child mortality rates are also high [7-10]. According to the Central Statistical Authority ([11]; 2011), Ethiopia has shown over the past few decades that as human numbers increase, the population-carrying capacity of the environment decreases. A high population growth rate increases the demand for resources and the rate at which these resources are exploited. It also states that the rationale for the 1993 Ethiopian National Population Policy was to address the imbalances between population growth and its natural resource base, given limited progress in productivity. In the absence of substantial migration and at any given mortality level, fertility is the most dynamic element in determining the rate of natural population growth [11,12]. In Ethiopia, urban fertility has been declining since 1990, suggesting an earlier start of fertility transition in urban Ethiopia, while rural fertility has been struggling to improve. EDHS (2011) results also confirm this fact. The average fertility rate in rural areas is about 6 children per woman, while it is 2.4 children per woman in urban areas. This high reproductive performance among the rural population may be explained by early and universal marriage, the high social and economic value attached to children, the low level of infertility, the depressed status of women, and the low use of contraceptives [13,14]. Similarly, information on fertility preferences provides insight into a couple’s attitude towards future childbearing, desired completed family size, the extent of unwanted and mistimed pregnancies, and the prevailing demand for contraception [15-19]. It also shows how fertility preferences vary depending on women’s socioeconomic, demographic, and cultural backgrounds. Change in women’s desired numbers of children is found to be a key determinant in the demand for family planning and the decline in fertility rate [20]. It is with this concept in mind that the investigators of this study wanted to correlate the promotion of after-child-birth-use of family planning, and desire to limit childbearing in Ethiopia. Postpartum amenorrhoea signifies the interval between childbirth and the return of menstruation. Many studies deal with the major socioeconomic, demographic, and cultural factors that influence married women’s desire to limit childbearing, especially in the rural areas of the study’s focus. Rapid population growth as a function of high fertility is observed mainly in rural areas, rather than urban areas [21-23]. Based on the review of literature, this study diversely focuses on the desire to limit the family size in predominantly sterilized, fecund, postpartum amenorrhoea, declared in-fecund, and/or menopausal cases of the study area. An important component of efforts to reduce health risks to mothers and children is increasing the proportion of babies that are delivered in health facilities. Unfortunately, only 10% of births in Ethiopia are delivered at a health facility (9% in a public facility and 1% in a private facility). Delivery that is assisted by skilled health service providers is another important intervention in reducing maternal mortality, but in Ethiopia, just 10% of births were assisted by a skilled service provider (4% by a doctor and 6% by a nurse or midwife) [13]. However, use of any contraceptive methods has increased to 29%, as noted in the EDHS 2011. Therefore, the authors are interested in seeing how the length and intensity of breastfeeding influences the duration of amenorrhoea, which offers protection from conception and thus future childbearing. Postpartum abstinence denotes the period between child birth and the time when a woman resumes sexual activity. None of the previous studies have described the linking factors behind the desire to limit childbearing among rural women. Therefore, this innovative study concerns itself with the significant factors that influence women’s desire to limit childbearing, and the factors that affect the desire for more children among rural married women. The general aim of this study is to investigate the major socioeconomic, demographic, and cultural factors that influence married women’s desires to limit childbearing. Moreover, the specific objective is to examine the desire to limit family size, along with cases of sterilized, fecund, postpartum amenorrhoea, declared in-fecund and menopausal women within the study area.

## Methods

The study is based on the analysis of secondary data obtained from the 2011 Ethiopian Demographic and Health Survey (EDHS). There are different data files such as births, household, individuals, children, males, household members, and couples’ records. This study is concentrated on couples because we need to know about married people’s desire to limit their family size. The total number of respondents was 6,745 (78.3% rural and 21.7% urban), with 93.6% of them being currently married and 6.4% of them living with a partner. These women were asked questions on the background characteristics such as age, education and media exposure, birth history, child mortality, knowledge and use of family planning methods, fertility preferences, antenatal, delivery and postnatal care, breastfeeding and infant feeding practices, vaccinations, childhood illnesses, marriage and sexual activities, women’s work, husband’s background characteristics, awareness and behaviours regarding AIDS and other sexually transmitted infections and adult mortality, including maternal mortality.

This study attempts to examine the quality of fertility data, using age-specific marital fertility rate, average parity, and proportion of children dead. Since the main objective is to find out the major determinants of desire to limit childbearing, data must be examined on fertility preferences of the study area. This was done using the consistency of the constructed variable, which shows whether ideal family size exceeds, equals, or is less than the actual number of children, and the respondents’ desire for more children. The bivariate analysis, including a chi square test is used to analyse the association between the two dependent variables, *ideal number of children* and *desire for more children*. The analysis examines two basic dependent variables such as desire for more children and desire to limit childbearing. The bivariate, ANOVA, and multivariate analyses were used to analyse the association and examine the relationship between the dependent and independent variables.

## Results

Table 1 summarizes the results on women who desire to limit childbearing, postpartum amenorrhoea, sterilization, and in-fecundity in 2011 Ethiopia. Overall, the desire to limit family size is 31.5%, with some variation between regions: the highest in SNNP is about 4 out of every 10 currently married women (38.9%), and the lowest is Somali with 9.1%. In Addis Ababa, 8 out of 393 (2.0%) currently married women were sterilized when the survey was conducted, followed by SNNP with five out of 928 (0.5%) and Gambela with two out of 409 (0.5%). B & G, with one out of 628 (0.2%), and Dire Dawa with one out of 410 (0.2%), are the regions where the percentage of sterilized women are the lowest. None of the currently married women in Affar and Somali used sterilization for contraceptive methods. The maximum postpartum amenorrhoea recorded in Oromiya was 366 out of 1081 (5.4%) among currently married women, and the lowest was Addis Ababa with 54 out of 393 (0.8%). The highest level (percentage) of women declared in-fecund was in Amhara (28 out of 999 [2.8%]), followed by B & G (15 out of 628 [2.4%]) and Addis Ababa (9 out of 393 [2.3%]).

**Table 1:** Distribution of currently married women’s desire to limit, sterilized, postpartum amenorrhea, declared in-fecund, in-fecund & menopausal by region in Ethiopia, 2011

**Region**	**No. of women**	**Desire to limit (no more children)**	**Sterilized (respondent or partner)**	**Fecund**	**Postpartum amenorrhoea**	**Declared in-fecund**	**In-fecund & menopausal**
Tigray	637	172 (27)	2 (0.3)	261 (3.9)	219 (3.2)	6 (0.9)	78 (1.2)
Affar	443	57 (13)	-	160 (2.4)	137 (2.0)	7 (1.6)	71 (1.1)
Amhara	999	376 (37.8)	4 (0.4)	460 (6.8)	293 (4.3)	28 (2.8)	166 (2.5)
Oromiya	1081	378 (35)	3 (0.3)	409 (6.1)	366 (5.4)	18 (1.7)	172 (2.6)
Somali	364	33 (9.1)	-	146 (2.2)	107 (1.6)	2 (0.6)	33 (0.5)
B & G	628	207 (33.1)	1 (0.2)	257 (3.8)	173 (2.6)	15 (2.4)	97 (1.4)
SNNP	928	359 (38.9)	5 (0.5)	362 (5.4)	314 (4.7)	4 (0.4)	104 (1.5)
Gambela	409	110 (26.9)	2 (0.5)	199 (3.0)	105 (1.6)	6 (1.5)	66 (1.0)
Harari	453	153 (33.8)	2 (0.4)	216 (3.2)	92 (1.4)	6 (1.3)	91 (1.3)
Addis Ababa	393	124 (31.6)	8 (2.0)	228 (3.4)	54(0.8)	9 (2.3)	79 (1.2)
Dire Dawa	410	150 (36.6)	1 (0.2)	173 (2.6)	100 (1.5)	7 (1.7)	74 (1.1)
**Total**	**6745**	**2119 (31.5)**	**28 (0.4)**	**2871 (42.6)**	**1960 (29.1)**	**108 (1.6)**	**1031 (15.3)**

Note: Based on the couples’ record 2011 Ethiopian Demographic and Health Survey data was estimated.

The average fertility preference of fecund women is placed between 1.62 and 1.69 (Table 2). The mean fertility preference of pregnant women falls between 1.58 and 1.70. The average of fertility preference of women in postpartum amenorrhea falls between 1.62 and 1.71. We can conclude from Table 2 that the 95% - confident interval of the mean of desire for more children among fecund women falls between 2.69 and 2.81. The desire for more children among pregnant women falls between 2.88 and 3.07. The mean desire for more children among women in postpartum amenorrhea falls between 2.86 and 2.99. The average desire for more children for in-fecund/menopausal women shows a mean between 3.36 and 3.61. Furthermore, the ideal number of children for pregnant women falls between 15.92 and 20.07. Remarkably, the ideal number of children for women in postpartum amenorrhea falls between 17.13 and 19.92. The ideal number of children for in-fecund/menopausal women falls between 13.49 and 16.92. Women are considered to be insusceptible to pregnancy if they are not exposed to the risk of conception, either because their menstrual period has not resumed since giving birth or because they are abstaining from intercourse after childbirth. ANOVA results show that the mean duration of postpartum amenorrhea is much shorter, just nearly 3 months. The mean duration of amenorrhea among women who gave birth in the five years preceding the survey is 16 months. Taken together, these two factors show that the mean duration of postpartum insusceptibility to pregnancy is 19 months.

**Table 2:** One-way ANOVA presents fertility preferences by exposure (fecund) in Ethiopia 2011

**Preferences**	**No. of women**	**Mean**	**Std. deviation**	**Std. error**	**95% CI for mean**		**F**	**Sig**
					**Lower**	**Upper**		
*Fertility preference*							90.43	0.000
Fecund	2865	1.66	.96	.02	1.62	1.69		
Pregnant	881	1.64	.90	.03	1.58	1.70		
Postpartum amenorrheic	1956	1.66	.94	.02	1.62	1.71		
In-fecund, menopausal	1030	2.21	1.28	.04	2.14	2.29		
Total	6732	1.74	1.02	.01	1.72	1.77		
*Desire for more children*							50.64	0.000
Fecund	2865	2.75	1.66	.03	2.69	2.81		
Pregnant	881	2.97	1.41	.05	2.88	3.07		
Postpartum amenorrheic	1956	2.92	1.51	.03	2.86	2.99		
In-fecund, menopausal	1030	3.49	2.04	.06	3.36	3.61		
Total	6732	2.94	1.67	.02	2.90	2.98		
*Ideal number of children*							6.57	0.000
Fecund	2868	15.10	28.33	.53	14.06	16.14		
Pregnant	882	18.00	31.39	1.06	15.92	20.07		
Postpartum amenorrheic	1959	18.53	31.56	.71	17.13	19.92		
In-fecund, menopausal	1031	15.21	28.06	.87	13.49	16.92		
**Total**	**6740**	**16.49**	**29.71**	**.36**	**15.78**	**17.20**		

Note: Based on the couples’ record 2011 Ethiopian Demographic and Health Survey data was estimated.

In Table 3 & Table 4, the multivariate analysis results of desire of more children (gross and net effects) in rural areas showed that religion, education, annual livestock and land unit, sex composition, and number of living children are statistically significant and associated with the desire for more children. Women with an education level ranging from grades 1-4 were found to be 3.08 times more likely to desire more children, though the level of significance was reduced; and women with medium durations of marriage were 72.8% (OR = 0.28) less likely to desire more children, compared to women in the reference category (short duration). In the same way, the odds of women with high durations of marriage desiring more children were 83.6% (OR = 0.16) lower than for the reference category. Women with equal numbers of sons and daughters were found to be 75.4% (OR = 0.25) less likely to desire more children, compared to women with more sons than daughters. Annual livestock and land unit, duration of marriage, sex composition of living children, number of living children, and expected economic value of children are associated with the ideal number of children (gross and net effects) in rural Ethiopia: women in the medium and high annual livestock and land unit categories reported larger ideal numbers of children than those in the reference category, and the odds of women in medium and long durations of marriage having large numbers of children were 2.11 and 3.27 times higher than for women with short durations of marriage.

**Table 3:** Results of multivariate analysis (odds ratio) for couples who desire more children in rural Ethiopia, 2011

	**Model I (gross effect)**			**Model II (net effect)**		
	**B**	**S.E.**	**Exp (β)**	**B**	**S.E.**	**Exp (β)**
*Religion*						
Orthodox Christian *(Ref)*						
Muslim	1.76	0.32	5.84***	2.80	0.41	16.42***
*Education*			***			*
Illiterate *(Ref)*						
Only read and write	-0.37	0.30	0.69	-0.42	0.48	0.66
1-4	1.10	0.30	2.99***	1.12	0.39	3.08**
5+	0.57	0.32	1.77	0.471	0.45	1.60
*Exposure to media*						
Never *(Ref)*						
Some times	-0.16	0.15	0.86	-0.23	0.21	0.81
Frequently	-0.50	0.31	0.61	-0.47	0.48	0.63
*Knowledge of FP methods*						
Low *(Ref)*						
Medium	-0.10	0.39	0.90	1.01	0.66	2.74
High	0.049	0.42	1.05	1.16	0.71	3.20
*Livestock and land unit*			***			**
Low *(Ref)*						
Medium	-0.26	0.17	0.77	0.67	0.24	1.95**
High	-0.86	0.17	0.42***	0.66	0.25	1.93**
*Age group*			***			***
15-19 *(Ref)*						
20-34	-1.82	0.53	0.16**	0.38	0.67	1.45
35-49	-3.56	0.54	0.03***	-0.65	0.71	0.52
*Duration of marriage*			***			**
Short *(Ref)*						
Medium	-1.28	0.19	0.28***	-0.41	0.26	0.67
Long	-1.81	0.19	0.16***	0.47	0.34	1.59
*Living children composition*			*			***
Sons > daughters *(Ref)*						
Sons = daughters	-0.37	0.19	0.69	-1.40	0.26	0.25***
Sons < daughters	0.08	0.17	1.08	0.11	0.22	1.12
*Under five mortality*						
Yes *(Ref)*						
No	-0.32	0.16	0.73	0.07	0.22	1.07
*Number of living children*			***			***
0-2 *(Ref)*						
3-4	-0.81	0.19	0.45***	-0.95	0.27	0.39***
5+	-2.41	0.20	0.09***	-2.85	0.37	0.06***
**Constant**				-0.01	0.94	0.99

Note: Based on the couples’ record 2011 Ethiopian Demographic and Health Survey data was estimated.

*** = P < 0.001; ** = P < 0.01; * = P < 0.05 (***,**,*Indicate level of significance at specified level); Ref = reference category.

**Table 4:** Multivariate analysis results (odds ratio) of reported ideal number of children in rural Ethiopia, 2011

	**Mode I (gross effect)**			**Model II (net effect)**		
	**B**	**S.E.**	**Exp (β)**	**B**	**S.E.**	**Exp (β)**
*Religion*						
Orthodox Christian^@^						
Muslim	1.08	0.23	2.96***	0.57	0.31	1.77
*Education*			**			
Illiterate^@^						
Only read and write	0.83	0.30	2.30**	0.60	0.37	1.82
1-4	0.47	0.25	1.59	0.44	0.31	1.56
5+	-0.63	0.35	0.53	-0.09	0.42	0.91
*Exposure to media*						
Never^@^						
Some times	0.17	0.16	1.18	-0.18	0.20	0.83
Frequently	0.32	0.31	1.38	0.20	0.42	1.22
*Knowledge of FP*			*			
Low^@^						
Medium	-0.46	0.39	0.63	-0.60	0.47	0.55
High	-0.98	0.43	0.38*	-0.95	0.55	0.39
*Livestock and land*			***			***
Low^@^						
Medium	0.65	0.19	1.91***	0.62	0.23	1.86**
High	1.16	0.18	3.19***	0.91	0.24	2.48***
*Age group*			***			***
15-19^@^						
20-34	0.37	0.33	1.43	0.06	0.69	1.06
35-49	1.18	0.34	3.27***	0.99	0.72	2.68
*Duration of marriage*			***			***
Short^@^						
Medium	0.64	0.19	1.89**	0.75	0.23	2.11**
Long	1.33	0.19	3.77***	1.19	0.27	3.27***
*Living children composition*						
Sons > daughters^@^						
Sons = daughters	0.15	0.20	1.16	0.44	0.24	1.55
Sons < daughters	0.39	0.17	1.48*	0.44	0.2	1.56*
*Number of living children*			***			
0-2^@^						
3-4	1.39	0.20	4.02***			
5+	1.71	0.20	5.50***			
*Economic value of children*			***			***
High cost^@^						
Medium benefit	0.76	0.25	2.17**	1.52	0.31	4.59***
High benefit	0.85	0.16	2.33***	1.73	0.232	5.61***
**Constant**				-1.05	0.85	0.35

Note: Based on the couples’ record 2011 Ethiopian Demographic and Health Survey data was estimated.

Notes: *** = P < 0.001; ** = P < 0.01; * = P < 0.05 (***,**,*Indicate level of significance at specified level).

^@^ = reference category.

## Discussion

Although more than half (57%) of currently married women aged 15-49 reported that they want more children, 38% want to wait at least two years or more before having their next child. These women can be considered potential contraceptive users for spacing births. Just 2% of women consider themselves to be in-fecund. The desire for more children is related to the number of living children women already have. Nine in every ten married women with no children want to have a have a child, with 55% expressing the desire to have a child soon and 34% wanting to delay having a child for at least two years. For women with one or more living children, the desire to stop childbearing altogether increases with the number of children. For example, 9% of married women with one child report that they want no more children, compared to 69% of women with 6 or more children. Nevertheless, women with 5 children (37%) or 6 or more children (22%) want to have another child. Unfortunately, for currently married women of reproductive age, the desire to space births has not changed considerably over time. There is also significant regional variation in the desire to limit childbearing, ranging from 11% in the Somali region to 41% in the SNNP region. As women’s education increases, their reported desire to have no more children decreases. For example, 41% of women with no education desire to limit childbearing, compared to 32% of women with primary education.

The mean ideal number of children is 4.3 for all women and 4.9 for currently married women. Among all women, the mean ideal number of children increases with the number of living children, from 3.3 for women with no children to 6.2 for women with six or more children. In the study area, men, on average, prefer to have larger families than women consider ideal. Among all men, the ideal family size is 4.8 children, while it is 5.9 children among married men. Similarly, currently married men with 6 or more children want more children (8.6 children) than the number that married women want (6.2 children). This case is clearly confirmed with the ANOVA test. The results show a rather low use of sterilization as a contraceptive method of choice among Ethiopian women. Sterilization is a permanent type of contraceptive that most women will not readily consider, due to the fact that they may later decide to have one or two additional children. There is a low use of contraceptives among married women in sub-Saharan Africa, even though many countries have implemented programs to increase the uptake of contraceptives as means of decreasing rapid population growth and high fertility in the continent [24]. The desire to limit family size is below 50%, suggesting that married women aim for larger family sizes than the ones they currently have. For instance, the results show that women with 5 children (37%) or 6 or more children (22%) want to have another child – specifically, over two-fifths (42.6%) of fecund women reported that they desire more children, and 13.1% of pregnant women desire more children. The current situation has some serious implications for the development and future distribution of resources among the growing population. A previous study in Ethiopia in 2008 (using the 2005 EDHS) found that three regions, Addis Ababa, Amhara, and Oromia, had more than two-fifths of women desiring to limit childbearing. Compared to these 2008 findings, the current study findings showed a decrease in the percentages of women desiring to limit childbearing in these three regions (at less than 40%). A study in Nigeria found that certain myths prohibit women from seeking family planning, such as the belief that contraceptive use is associated with women who are “promiscuous” [25]. These beliefs can be attributed to many rural areas in Africa, where women still hold onto cultural and traditional beliefs that make them sceptical of change.

The multivariate results showed that various factors influence the desire for more children in rural areas of Ethiopia. These factors include religion, education, annual livestock and land unit, sex composition, and number of living children. Regarding the number of living children, one might argue that women who have lost a child will be more likely to desire more children as means of filling the gap brought on by the loss of that particular child. To support this claim, the results show that women who have less than three living children are more likely to desire additional children. Therefore, we can deduce that having fewer living children is negatively correlated with a couple’s likelihood to limit childbearing. Concerning religion, the study findings showed that Muslim couples are more likely to desire more children than Orthodox Christians. A study with similar results found that in Kenya and Tanzania, Muslim women desire more children than Christians [26]. Furthermore, the results showed that factors that correlate with ideal number of children include annual livestock and land unit, duration of marriage, sex composition of living children, number of living children, and expected economic value of children. Regarding annual livestock and land unit, women in the medium and high annual livestock and land unit categories reported larger ideal numbers of children than those in the low category. Also, women with an educational attainment less than grade one were found to have a larger ideal number of children, compared to those who are in the reference category, i.e., illiterate women (as well as those who have attained grade one or higher). Another interesting finding is that women who attach a “high-benefit” economic value to children have a larger reported ideal number of children (i.e., family size) than women who perceive children to be a high cost. This finding is interesting due to its suggestion that couples view their children as an investment and that these children are due for pay-up when their parents reach old-age. In African traditions or cultures, it is almost always expected that once the child grows older, he/she will be obligated to financially pay back (i.e., support) the parent(s) for his/her upbringing. In the same token, it can thus be asserted that couples (or parents) who attach a high value to having children will aim for larger families who will later fit the role of being a provider and caregiver to the parents in old age. The quantitative results suggest that there are several determinants of health care application in the country. These determinants have an influence on the frequency with which women use the available quality health care services, especially maternal health care [4]. Therefore, this study promotes women’s health by determining priorities, delineating family planning use after child birth, and informing evidence-based interventions founded on the basic and insightful information provided on social capital and the status of the health of women. This research will also be significant to policy-makers, helping them to enhance quality health care services and promoting antenatal care, postnatal care, and maternal health education programmes for women in Ethiopia.

## Conclusion

Based on the study’s findings, the mean duration of postpartum amenorrhea is just 3 months. Appropriate factors show that the mean duration of postpartum insusceptibility to pregnancy is 19 months, which is too short. It is clearly visible that antenatal and post natal care service is very weak/poor in Ethiopia. Therefore, there is an urgent need to amend/promote antenatal and postnatal care, including an increase in breastfeeding to at least 24 months. There is also a need to enthusiastically promote institutional delivery as well as health service providers’ field visits, especially compulsory ANM visits in rural areas. Proper monitoring and evaluation is equally compulsory. Additionally, as per the study’s findings, there is a difference in the ideal number of children among rural women who expect high costs, medium benefits, and high benefits of childbearing. Women with an equal numbers of sons and daughters were less likely to desire more children, compared to women with more sons than daughters. Finally, achievable resolutions include increasing females’ ages at marriage, avoiding unwanted teenage pregnancies, completely eradicating home delivery, and inspiring young people to use modern methods to achieve Millennium Development Goals 4 & 5.

## Consent

Consent was not necessary because the study used secondary data from the Ethiopian Demographic and Health Survey 2011 Email: info@measuredhs.com, Internet: (http://www.measuredhs.com). All data were de-identified. If additional information about the 2011 EDHS may be obtained from the Central Statistical Agency, Email: csa@ethionet.et.

## Competing interests

The authors declare that they have no financial or non-financial competing interests in relation to this manuscript.

## Authors’ contributions

All authors have made substantial contributions to conception, acquisition and interpretation of data. They have participated in drafting the article and AS extensively reviewed and revised the article. All authors read and approved the final manuscript.

## Author’s information

Professor A Sathiya Susuman has an MA, MPhil in Population Studies and a PhD in Demography. He has specialized in the social science research area of demographic analysis and reproductive health. His specific research area is fertility, mortality, gender, reproductive health and public health. He has published several articles in reputed journals.

Aristide Bado is a doctoral research fellow in the Dept. of Statistics and Population Studies, University of the Western Cape, Cape Town South Africa.

Yishak Abraham is a post graduate student in the Dept. of Statistics and Population Studies, University of the Western Cape, Cape Town South Africa.
